# Pharmacological Interventions for REM Sleep Behavior Disorder in Parkinson's Disease: A Systematic Review

**DOI:** 10.3389/fnagi.2021.709878

**Published:** 2021-08-13

**Authors:** Junqiang Yan, Anran Liu, Jiarui Huang, Jiannan Wu, Ruile Shen, Hongxia Ma, Jianxue Yang

**Affiliations:** ^1^Key Laboratory of Neuromolecular Biology, The First Affiliated Hospital of Henan University of Science and Technology, Luoyang, China; ^2^Department of Neurology, The First Affiliated Hospital, College of Clinical Medicine of Henan University of Science and Technology, Luoyang, China; ^3^School of Nursing, The First Affiliated Hospital of Henan University of Science and Technology, Luoyang, China

**Keywords:** Parkinson's disease, rapid eye movement, drugs, systematic review, sleep

## Abstract

To review the therapeutic effects of drugs on REM sleep behavior disorder (RBD) in Parkinson's disease (PD) by searching the MEDLINE/PubMed, Embase, Cochrane, and CBM databases. According to the inclusion and exclusion criteria, studies were included after excluding duplicate data. We evaluated the safety and efficacy of pharmacological intervention to improve RBD in patients with Parkinson's disease (PD-RBD). This systematic review mainly describes the drugs that can be used to treat PD-RBD patients. The results have shown that melatonin can be used as the first-line drug for PD-RBD, and clonazepam provides significant improvement on PD-RBD, androtigotine can be used as an alternative drug. However, further large-scale clinical trial studies are still needed to provide the best guidelines for the pharmacological treatment of PD-RBD.

## Introduction

Parkinson's disease (PD) is the second most common progressive neurodegenerative disease in elderly individuals over the age of 65 (Sherer et al., 2012). In addition to the typical motor symptoms, there are also non-motor symptoms such as constipation, dysphagia, cognitive impairment, and sleep disorders (McDonald et al., 2018) which seriously affect patients' quality of life (Chaudhuri and Schapira, 2009). Sleep disorders in PD mainly include insomnia, excessive daytime sleepiness (EDS), restless legs syndrome (RLS), and rapid eye movement (REM) sleep behavior disorder (RBD) (Chahine et al., 2017; Stefani and Hogl, 2021).

RBD is a sleep disorder characterized by dreams and physical activities during REM sleep. Most RBD patients have dream-related Violent behavior, which often results to injure themself or others. RBD can be divided into idiopathic RBD (iRBD) and secondary RBD (sRBD) according to different causes. The iRBD appears as an independent symptom without other accompanying symptoms; sRBD includes drug-induced, symptomatic and neurodegenerative diseases related RBD. IRBD is the most reliable clinical marker for pro-synucleinopathy, such as Parkinson's disease (PD) (Hogl et al., 2018). Patients may eventually develop neurodegenerative diseases after a few years or decades, and the risk of occurrence ranges from more than 30% at 5 years to more than 90% at14 years. Idiopathic RBD can be used as a pre-exercise biomarker for PD and about 20% of RBD occurred before PD and about 20% of cases had both Parkinson's disease and RBD and more than 50% of RBD occurred for several years after the clinically manifest of Parkinson's disease (Diaconu et al., 2021).

The incidence of RBD in PD patients is ~20–50% (Sixel-Doring et al., 2011; Romenets et al., 2012; Bugalho and Viana-Baptista, 2013). The main symptoms can vary from simple muscle tension to complex behavioral disorders (Gagnon et al., 2002; Schenck and Mahowald, 2002). Patients often yell, laugh and even have violent behavior in their sleep, which is usually discovered by their bed partners. Patients can often remember their vivid dreams and dream enactment when they wake up (Sforza et al., 1997; Olson et al., 2000). PD-RBD is generally observed and diagnosed by polysomnography (PSG) (Duchna, 2006). PD patients with RBD not only suffer from a decline in sleep quality, but also easily cause injuries to the patients themselves and their bed partners, increasing the risk of intimacy interruption and bed partner injuries (Postuma et al., 2012; Schenck et al., 2013a).

RBD not only affects sleep quality but also cognitive function in PD patients (Nomura et al., 2013a; Yarnall et al., 2013). Research by Schenck et al. showed that RBD is related to cognitive decline, and more than 80% of elderly patients with RBD develop Parkinson's disease or dementia (Schenck et al., 2013b). Compared with PD patients without RBD, the cognitive dysfunction (especially delayed memory function) of PD patients with RBD is more prominent (Zhang J. R. et al., 2016). A recent clinical study confirmed that there is a significant correlation between sleep efficiency and overall cognitive ability in patients with PD (Sobreira et al., 2019). Therefore, the clinical treatment of patients has become important and needed. This article mainly collects all relevant studies to analyse and evaluate the effectiveness and safety of drug interventions, and puts forward some suggestions and questions.

## Materials and Methods

### Search Strategy and Selection Criteria

This study used the search generators available in each database to search all relevant literature up to January 1, 2020 in the MEDLINE/PubMed, Embase, Cochrane, and CMB databases. The search method was based on the following terms: “Parkinson's disease” and synonyms and “rapid eye movement sleep behavior disorder” and related terms; the search commonly used acronyms for these phrases, and duplicate studies were excluded.

### Study Selection

The search results were independently evaluated by two reviewers, and differences were resolved through discussion. The inclusion criteria included the following: 1. Studies with crossover trials or open-label designs; 2. Studies that involved patients with Parkinson's disease; 3. The study targeted RBD among sleep disorders; 4. The treatment involved a single drug; and 5. Experiments were performed *in vivo*. The exclusion criteria were as follows: 1. Duplicate studies; 2. Studies that involved patients with diagnosis other than Parkinson's disease; 3. The studies sleep events related to PD other than RBD; 4. The treatment involved non-drug therapy or non-monotherapy; 5. Experiments were performed *in vitro*. In theory, we followed the PRISMA guidelines (Liberati et al., 2009) for a systematic review. However, due to the qualitative and non-quantitative research in this study, we did not further conduct bias risk assessment and data extraction synthesis.

## Results

**Figure 1:**
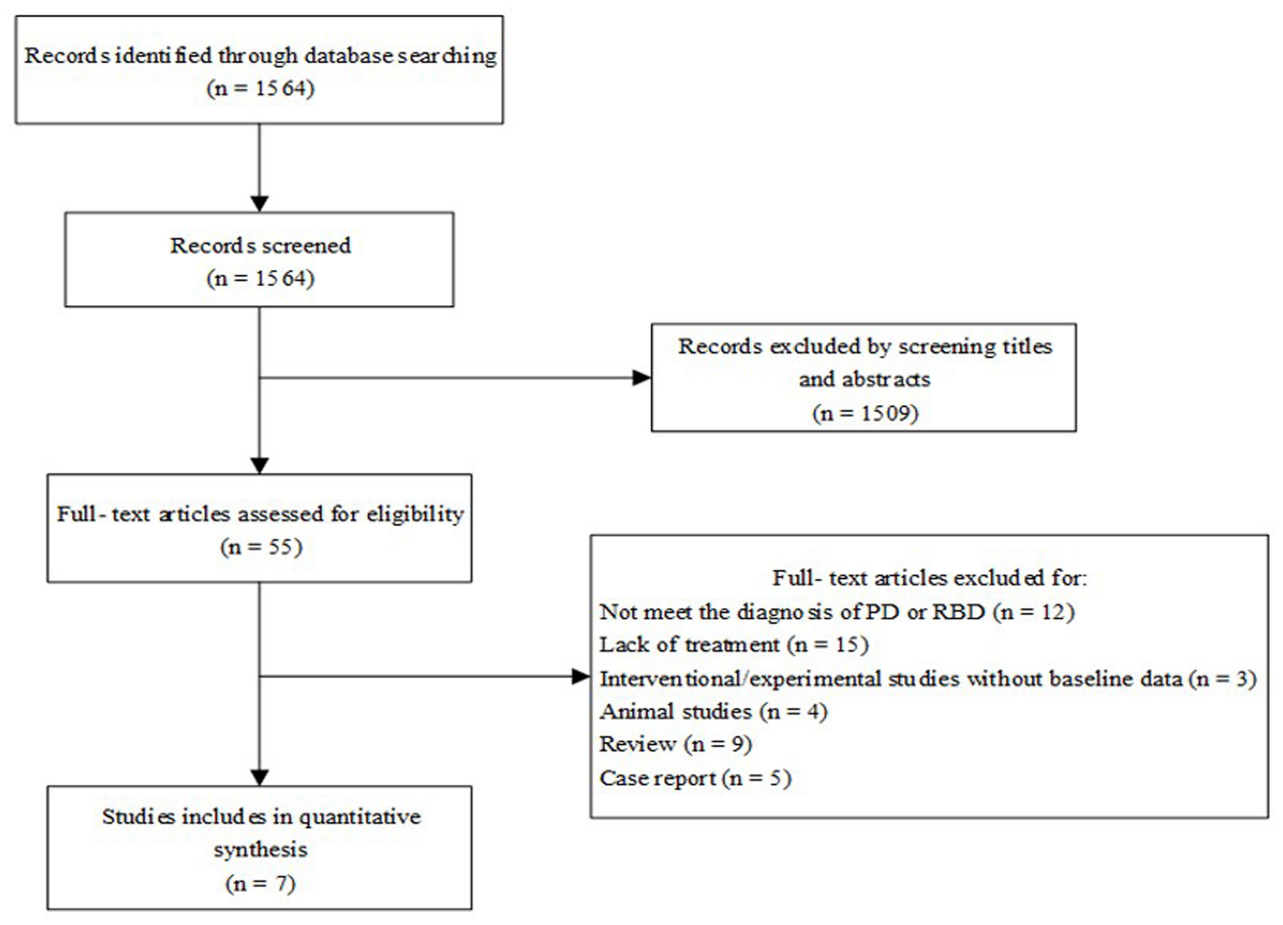
The PRISMA flow diagram.

Literature searches were conducted based on PRISMA's preferred reporting project guidelines (Figure 1) (Liberati et al., 2009). After duplicate studies were excluded, resulting in 1,564 articles. Based on the study's inclusion criteria, 55 related clinical drug studies were selected, of which seven clinical trials met the criteria.

### Clonazepam

Clonazepam has long been considered the first-line treatment for PD-RBD (Sforza et al., 1997; Olson et al., 2000; Schenck and Mahowald, 2002; Aurora et al., 2010) and has been widely used clinically (Seppi and Ray Chaudhuri, 2019). However, evidence for its effectiveness is based only on case reports and follow-up studies (Aurora et al., 2010; Li et al., 2016), and evidence for treatment in randomized controlled clinical trials is lacking. In 2013, the International RBD Study Group (IRBD-SG) published a consensus statement on the design of controlled clinical studies for RBD (Schenck et al., 2013a). Shin compared the overall clinical impression improvement score at the fourth week of using 0.5 mg clonazepam and placebo, and the results showed no significant difference (*p* = 0.253) (Shin et al., 2019). However, this experiment was mainly evaluated by the Clinical Global Impressions-Improvement (CGI-I) score, without further clarification with PSG. At the same time, research shows side effects (morning sedation, confusion, dizziness, and falls) which may limit the effectiveness of clonazepam, especially in the elderly and/or RBD that coexists with obvious neurodegenerative diseases (Anderson and Shneerson, 2009). The result remains to be demonstrated by more experiments.

### Melatonin

Clinical evidence has indicated that melatonin can be an effective adjuvant therapy for RBD in PD patients (Aurora et al., 2010). A study showed that after 4 weeks of treatment with 3–6 mg melatonin, RBD symptoms in 84% of PD patients significant improvement (Lyashenko et al., 2015). A randomized controlled study including 30 PD patients also showed that the extended release of 4 mg of melatonin did not significantly reduce PD-RBD symptoms (*P* = 0.92) (Gilat et al., 2020), but the study was mainly based on the Movement Disorder Society (MDS)-UPDRS questionnaire score, and the small sample couldn't detect group differences on secondary outcomes, and there were differences in the primary data at baseline level ADDIN EN.CITE (Gilat et al., 2020). In a multi-site, double-blind, placebo-controlled, crossover trial, 50 mg of melatonin improved sleep quality better than 5 mg (Dowling et al., 2005). Ramelteon, a melatonin receptor agonist, can significantly improve the RBD symptoms of PD patients compared with control group in a Multicenter Open Trial (*P* < 0.05) (Kashihara et al., 2016). But, the study mainly used the Japanese version of the RBD screening questionnaire to diagnose RBD, and did not use PSG, therefore, it had limitations. In another case report, PD-RBD patient was treated with ramelteon (8 mg/day before sleeping), PSG monitoring found that RBD symptoms were significantly improved (Nomura et al., 2013b). A meta-analysis in 2016 further demonstrated that melatonin showed significant improvements on sleep disorders in neurodegenerative diseases through nine randomized controlled trial (Zhang W. et al., 2016). The results of the latest 4-week randomized, double-blind, placebo-controlled pilot study showed that there was no difference between the iRBD patients receiving sustained-release melatonin and the placebo group. Although the study subjects were not PD-RBD, they didn't effect on results (Jun et al., 2019).

### Rotigotine

Wang et al. (2016) studied rotigotine transdermal patches for 7 months in PD-RBD patients through interviews with PD patients themselves and their families, the REM Sleep Behavior Disorder Questionnaire (RBDQ-HK) and video polysomnography (VPSG) measurements, and VPSG analysis showed that total sleep time (TST) and stage 1% were increased, and the PLMS index decreased (Wang et al., 2016). The results suggested that rotigotine can improve symptoms of RBD of PD patients. Pierantozzi et al. also designed a randomized, double-blind, placebo-controlled parallel experiment, and they found that rotigotine could significantly improve sleep efficiency of patients with PD through PSG, but the study lacked the definition of RBD (Pierantozzi et al., 2016).

### Rivastigmine

A double-blind crossover trial study (Di Giacopo et al., 2012) using rivastigmine at 4.6 mg/d showed a significant reduction in the frequency of RBD episodes recorded by bed partners (*P* = 0.027). However, four patients underwent multiple PSG tests, and REM sleep without atonia (RSWA) showed no significant changes (Di Giacopo et al., 2012).

### Pramipexole

In a prospective study, through bed partner recording and PSG monitoring, patients' PD symptoms improved, but RBD was not significantly improved (*P* > 0.05) (Kumru et al., 2008). This study was more accurate because of the combined subjective and objective detection methods, but the sample size was insufficient.

In addition, we briefly summarize the 55 articles in the query that involved drugs for treating sleep disorders but did not conform to the inclusion criteria, such as individual case reports or non-PD-RBD patients. This study provides additional information regarding these drug treatments as follows.

### Levodopa

Ozekmekçi et al. used levodopa at 460.3 and 320.3 mg/d in PD patients with RBD. Studies have shown that dopamine can improve the scores of UPDRS (Unified Parkinson's Disease rate Scale; Ozekmekçi et al., 2005). However, Wailke et al. did not find any improvement in REM sleep among PD patients' sleep status after taking 200 mg levodopa/carbidopa controlled-release tablet (CR) by PSG (*P* = 0.615) (Wailke et al., 2011). Tan et al.'s case report showed that RBD preceded PD in all three cases, and the three patients significantly improved their RBD after levodopa use, but without polygraph detection (Tan et al., 1996). There was a significant difference in subjective sleep symptoms (*P* = 0.082), six patients with PD-RBD received intestinal levodopa infusion after 6 months treatment (Zibetti et al., 2017).

### Cannabinoids

Cannabinoids (CBD) can also improve sleep quality and reduce sleep disorders (Kuhathasan et al., 2019). Clinical studies have shown that cannabinoids were beneficial for sleep disorders of PD patients, and the mechanism may be related to the distribution of cannabinoid receptors in the structure of the basal ganglia (Buhmann et al., 2019). In an observational study of four PD patients receiving CBD treatment, it was found that the frequency of RBD in patients was rapidly and significantly reduced, and there were no side effects (Chagas et al., 2014).

### Memantine

In a randomized controlled study (Larsson et al., 2010), sleep scale evaluation scores showed that 20 mg/d memantine reduced REM sleep behavior disorder that may occur in PD patients, but the specificity of PD-RBD was unclear.

## Discussion and Conclusions

We hereby conducted a systematic review of all relevant drug clinical trials to evaluate the safety and efficacy of drug treatment for PD-RBD. The latest consensus guidelines for the clinical management of PD non-motor symptoms published in 2020 mentioned that there is currently a lack of RCT (Randomized Controlled Trial) studies for PD-RBD treatment, and that clonazepam or/and melatonin can be used to treat PD-RBD (帕金森病非运动症状管理专家共识, 2020).

The results showed that melatonin and clonazepam can improve PD-RBD as current first-line drugs. Rotigotine can be used as an alternative or a monotherapy for the above two drugs, but more definitive clinical trial evidence is lacking. Askenasy thought that changes in dopamine receptor sensitivity in the substantia nigra and striatum during REM sleep may be responsible for sleep disorders (Askenasy, 1993). In addition, serotonin significantly decreased in the brains of PD patients, and levodopa activated dopamine receptors and reduced serotonin levels (Askenasy and Yahr, 1985), which may lead to improvement of PD-RBD, so dopamine therapy is promising to become an alternative to clonazepam. Rotigotine, a non-ergot dopamine agonist, has been demonstrated to be effective both an early monotherapy in PD (Watts et al., 2007) and adjuvant therapy for levodopa in advanced PD (Poewe et al., 2007). The mechanism of rotigotine to improve RBD may be through activation of dopamine D 1 receptors involved in the regulation of REM sleep (Wang et al., 2016). Santiago Pérez-Lloret concluded in his study that sleep changes in PD patients may be disrupted by the melatonin system, which can be associated with low levels of melatonin MT1 and MT2 receptor densities in the substantia nigra and amygdala in PD (Perez-Lloret and Cardinali, 2021). Clonazepam can improve RBD by activating glycine and GABA transport pathway (Brooks and Peever, 2011).

There is no reliable evidence to consider rivastigmine, memantine, cannabinoid, and other drugs, but it is still possible to consider them as adjuvant or even alternative drugs in the absence of significantly improved with first-line drug treatment. Cannabinoids (CBD) may regulate the changes in glutamic acid and other chemicals caused by decreased dopamine levels (Giacoppo et al., 2014; Gomez-Galvez et al., 2016; Stampanoni Bassi et al., 2017). Rivastigmine, as an acetylcholinesterase inhibitor, may be used in PD-RBD and acts on the cholinergic pathway in the context of pontine bulbar degeneration (Braak et al., 2003; Boeve et al., 2007). Videnovic et al. concluded that donepezil and quetiapine/clozapine can improve the symptoms of RBD, but these are individual case studies, and a large number of clinical studies are needed (During and Miglis, 2019).

In this study, according to the seven included literature studies, the results of a single trial show that (1) clonazepam and melatonin can currently be used as first-line drugs for the treatment of PD-RBD; (2) rotigotine can become a substitute for the above two drugs; and (3) rivastigmine, memantine, and cannabinoids may be effective for RBD in PD patients. According to the results of this systematic review, these drugs can improve the symptoms of PD-RBD and have been used clinically (Table 1). However, due to the insufficient sample size of these trials and the defects in evaluation methods, large-scale clinical trials are still needed for further confirmation.

**Table 1:** Main characteristics of the eligible studies.

**References**	**Design**	**Experimental intervention**	**Dosage**	**Duration of treatment**	**Measures**	**Findings**
Gilat et al. (2020)	Randomized, double-blind, placebo-controlled, parallel-group trial (*n* = 30)	Melatonin	4 mg	8 weeks	Video polysomnography (PSG); weekly CIRUS-RBD Questionnaire (wCIRUS-RBDQ); RBD Screening Questionnaire; Innsbruck RBD Inventory; RBD Questionnaire-Hong Kong; CGI; and International Parkinson and Movement Disorder Society (MDS)-UPDRS	4 mg of melatonin is well-tolerated, but not efficacious in ameliorating self-reported RBD in PD patients.
Lyashenko et al. (2015)	Open trial (*n* = 30)	melatonin	3–6 mg	4 weeks	Polysomnography; RBD Screening Questionnaire (RBDSQ); Parkinson Disease Sleep Scale (PDSS)	84% of patients reported reduction in RBD symptoms.
Shin et al. (2019)	Randomized, double-blind, placebo-controlled trial (*n* = 40)	Clonazepam	0.5 mg	4 weeks	Clinical Global Impressions-Improvement (CGI-I) scores	Both clonazepam and placebo tended toward improvements on RBD symptoms in patients with PD.
Kashihara et al. (2016)	A multi-center open trial (*n* = 24)	Ramelteon	8 mg/d	12 weeks	Japanese version of the RBD Questionnaire (RBDQ-JP); PD Sleep Scale Version-2 (PDSS-2); Unified PD Rating Scale (UPDRS)	The RBDQ-JP score was markedly reduced after the initiation of ramelteon treatment, ramelteon markedly improved RBDQ-JP scores, as well as UPDRS part III and PDSS-2 scores in patients with PD with RBD.
Di Giacopo et al. (2012)	Double-blind, crossover pilot trial (*n* = 12)	Rivastigmine	4.6 mg/d	3 weeks (T1), 3 weeks (T2)	RBD episode frequency reduction according to the bed partner's diary	Mean frequency of RBD episodes was significantly lower with rivastigmine treatment than with placebo (*Z* $\frac{1}{4}$ = 2.207; *P* $\frac{1}{4}$ = 0.027).
Kumru et al. (2008)	Prospective study (*n* = 11)	Pramipexole	0.54 mg/d	3 months	Video polysomnography (VPSG)	In PD patients, pramipexole improved parkinsonism but did not modify RBD-related symptoms and objective video-polysomnographic abnormalities.
Wang et al. (2016)	Prospective open-label study (*n* = 11)	Rotigotine patches	2–16 mg/d	7 months	Patient and bed partner interviews; a validated evaluation scale (REM sleep behavior disorder questionnaire-Hong Kong, RBDQ-HK) video polysomnography (VPSG)	Rotigotine improved parkinsonism and subjective sleep quality in PD patients with RBD.

## Data Availability Statement

The original contributions presented in the study are included in the article/supplementary material, further inquiries can be directed to the corresponding author/s.

## Author Contributions

AL and JW were involved in the execution. RS and JYang were involved in the statistical analysis and manuscript preparation. HM and JH were involved in analyzing the data. JYan was involved in the research project (conception, design, writing, and organization). All authors contributed to the article and approved the submitted version.

## Conflict of Interest

The authors declare that the research was conducted in the absence of any commercial or financial relationships that could be construed as a potential conflict of interest.

## Publisher's Note

All claims expressed in this article are solely those of the authors and do not necessarily represent those of their affiliated organizations, or those of the publisher, the editors and the reviewers. Any product that may be evaluated in this article, or claim that may be made by its manufacturer, is not guaranteed or endorsed by the publisher.
